# Pain in Some Foot Cases—III

**Published:** 1910-11-19

**Authors:** 


### ORTHOPÆDICS.

### PAIN IN SOME FOOT CASES.—III.

Acute cases of suppurative arthritis rarely come under the notice of the orthopædist: they go to the general surgeon and he treats them, leaving the orthopædist to bear the full responsibility of looking after and dealing with the results of his treatment! There are many cases of arthritis of a chronic nature affecting the foot which come primarily for the cure or improvement of a joint trouble, and in nearly all of them pain is a frequent symptom. With the osteo-arthritic variety we have already dealt. There remain, however, the syphilitic, gonorrhœal, rheumatic, and specific forms of rarer occurrence, such as the influenzal or pneumococcal types. In all these varieties, which, so far as pain is concerned, may be classed under the heading of specific joints, the foot is not so commonly affected as the knee or hip, and pain in the foot is certainly never so severe as in hip cases. In the syphilitic and rheumatic forms it may be fairly acute in its intensity, varying in severity with the posture or movements of the patient, while in the other varieties it is very rarely complained of. Pneumococcal infections of the tarsal and ankle joints seem to be almost painless; and certainly in this, as well as in the influenzal and gonorrhœal cases, the intensity of the pain is by no means a trustworthy index as to the severity of the lesion and the pathological changes taking place within the joint.

### Pain.

The subject of treatment of these varieties is too large to be here discussed, where we are mainly concerned with one symptom only —namely, pain. In the syphilitic cases, where the pain is aggravated by the hyperæmia induced by the warmth of the bedclothes, and is therefore said to be worse at night, potassium iodide, especially when given in combination with mercury, acts very beneficially. Indeed, one of the first effects of the administration of iodides to a syphilitic patient complaining of arthritic pains is the marked improvement that follows in the subjective symptoms. As most of the foot cases occur in young individuals, it is important to get rid of the pain as early as possible, and certainly no proper orthopædic treatment, calculated to cure or improve a deformity, can be successfully applied while the foot is acutely sensitive and tender. These cases, therefore, are in an entirely different category from those in which the pain is definitely due to the deformity, and vanishes when that deformity is carefully corrected. The treatment must be at first wholly directed to the pain, and although the deformity—or, rather, the possible results that may occur from the specific infection so far as the joint is concerned, should never be lost sight of, it is the pain that should receive primary attention. The practitioner will find that the iodides are his best standby in the luetic cases: inunctions, local massage, or the hyperæmic treatment cannot take their place In rheumatic cas[illegible] the salicylates, while ordinarily very effective, sometimes fail singularly in cases where the tarsal joints are affected. Then the drug must be pushed and local treatment must be adopted. Bier's treatment is often very efficacious in easing the swollen and painful rheumatic joint, while aids to the patient's comfort are to be found in wrapping the limb in warm cottonwool, packing ice round it, elevating it, or simply applying boracic or lysol fomentations, or, better still, putting the whole foot in a bicarbonate of soda solution bath at a temperature as high as can be conveniently borne. Sometimes a case that has proved obstinate to warm applications reacts very favourably to ice-bags or *vice versa*, but in every case where the salicylates appear to act slowly in controlling the pain it is worth while to try the effect of the Bier bandage.

There are some cases of flat-foot in which the pain complained of is apt to be put down as rheumatic. Here the salicylates are often entirely useless and the iodides equally so. The acute flat-foot cases are not included in this class; in these proper attention must be paid to the underlying primary cause, in the others an accurately fitted inset will usually relieve the pain.

### Old Paralytic Cases.

It is not generally remembered that in old paralytic foot cases pain may be complained of very bitterly by the patients. Such patients are, of course, children or young adults, and at first, especially if he overlooks the fact that it is not an uncommon symptom, the practitioner is apt to regard the complaints as exaggerated and to minimise the pain. It is certain that such pains in patients who have suffered from infantile paralysis are really very severe, and it is the orthopædist's duty to devote proper consideration to them. While the wearing of apparatus may do much to ease and relieve the patient, it is not always successful, and in any case it must be actively supplemented by massage and tonic treatment. Electrical treatment does not appear to relieve the pains, and had much better be dropped until no subjective sensations are complained of. Massage, however, is a valuable auxiliary. The pain is very stubborn and is associated with active movements of the limb. It usually is paroxysmal, and in the foot is chiefly felt across the dorsum and along the inner side of the instep. Locally hot applications (dry heat) do good in such cases, while internally small doses of the bromides appear to act the best.

Finally, although we have by no means exhausted the catalogue of the various pains that may be met with in foot cases, a word must, in conclusion, be said about the affection commonly known as painful ankle. This is a variety of tarsal pain which has many points of similarity with the metatarsalgia described by Morton and usually associated with his name, but it is more commonly felt at the back of the heel instead of over the dorsum of the foot. It is most commonly due to the presence of a small exostosis on the os calcis which must be removed before permanent relief can be obtained. For this a small surgical operation, which is easily done under a local anæsthetic, is necessary. Palliative measures and local applications are useless and should not be attempted in cases where the diagnosis (which may be considerably helped by the *x*-rays) is clear.

